# Different outcome in node-positive breast cancer patients found by axillary ultrasound or sentinel node procedure

**DOI:** 10.1007/s10549-017-4342-1

**Published:** 2017-06-27

**Authors:** Nicole C. Verheuvel, Adri C. Voogd, Vivianne C. G. Tjan-Heijnen, S. Siesling, Rudi M. H. Roumen

**Affiliations:** 1 Department of Surgery, Máxima Medical Center, PO Box 7777, 5500 MB Veldhoven, The Netherlands; 2 Department of Epidemiology, School of Oncology and Developmental Biology (GROW), Maastricht University Medical Centre, Maastricht, The Netherlands; 3 Department of Research, Netherlands Comprehensive Cancer Organisation (IKNL), Utrecht, The Netherlands; 4 Department of Medical Oncology, School of Oncology and Developmental Biology (GROW), Maastricht University Medical Centre, Maastricht, The Netherlands; 5 Department of Health Technology and Services Research, MIRA Institute for Biomedical Technology and Technical Medicine, University of Twente, Enschede, The Netherlands

**Keywords:** Ultrasound, Sentinel node, Breast cancer, Survival

## Abstract

**Background:**

The Z0011 trial initiated a paradigm shift in the axillary treatment of breast cancer patients with a positive sentinel lymph node biopsy (SLNB), disregarding patients with a positive ultrasound-guided lymph node biopsy (UGLNB). We examined whether relevant differences exist between these patients to determine if the conclusions of the ACOSOG Z0011 trial are applicable to UGLNB-positive patients.

**Methods:**

Patients diagnosed with invasive breast cancer in the Netherlands between January 2008 and December 2014 were selected from the Netherlands Cancer Registry.

**Results:**

A total of 11,820 cases were included: 9149 cases in the SLNB group and 2671 in the UGLNB group. Multivariate analyses showed that UGLNB-positive patients were older (*p* < 0.001), more likely to have a poorly differentiated tumor (*p* < 0.001), had a negative hormone receptor status (*p* < 0.001), and more often had extensive nodal involvement (*p* < 0.001). However, they were less likely to undergo adjuvant radiation (*p* = 0.004) or systemic therapy (*p* < 0.001). Even after adjusting for these factors, UGLNB-positive patients had a worse overall survival (HR = 1.38; 95% CI 1.23–1.56) than SLNB-positive patients.

**Conclusion:**

This nationwide retrospective study shows that young patients found positive by UGLNB have less favorable disease characteristics and a worse prognosis compared to patients with a positive SLNB. Selection by ultrasound plays an important role when axillary treatment strategies are considered. Hence, the conclusions of the Z0011 trial cannot unconditionally be applied to patients with a positive UGLNB.

## Introduction

Determining the axillary lymph node status is still an important element in the diagnostic work-up of patients with invasive breast cancer. This is mostly done by ultrasound-guided lymph node biopsy (UGLNB) or sentinel lymph node biopsy (SLNB) [1]. Until recently, a positive lymph node was immediately followed by an axillary lymph node dissection (ALND) to obtain locoregional disease control and to determine the need for adjuvant systemic treatment [1–4]. However, multiple studies have shown that in 40–70% of the patients with a positive SLNB the remaining non-SLNs do not contain any metastases [5–7]. As a consequence, the indication for adjuvant treatment has become increasingly dependent on other prognostic factors, such as age, tumor size, tumor grade, hormone and HER2 receptor status, and gene expression profiles [1, 8–10]. These developments raised questions on the necessity of performing an ALND in patients with a positive SLNB [6, 11].

Results from the American College of Surgeons Oncology Group (ACOSOG) Z0011 trial initiated a paradigm shift in the management of invasive breast cancer. This study showed that an ALND can be safely omitted in patients with a positive SLNB, treated with breast-conserving surgery (including radiotherapy) and adjuvant systemic therapy, even after a follow-up of nearly 10 years [12, 13]. However, UGLNB-positive patients were excluded in this study. In order to assess whether the conclusions of the Z0011 trial can also be applied to these patients, we have previously conducted a study among 302 node-positive patients to examine whether UGLNB-positive patients were comparable to SLNB selected positive patients with respect to important clinicopathological factors and prognosis. That study showed that UGLNB-positive patients had worse tumor characteristics and consequently had a worse disease-free and overall survival compared to patients with a positive SLNB [14].

However, these results were based on a small patient population treated in only one non-academic teaching hospital. We therefore perform this study in a nationwide population-based dataset containing data of breast cancer patients *without palpable lymphadenopathy* to examine whether relevant differences in disease characteristics and prognosis exist between patients selected by UGLNB versus SLNB to determine if the conclusions of the ACOSOG Z0011 trial are applicable to UGLNB-positive patients.

## Patients and methods

This population-based study included patients diagnosed with clinical stage *T*_1_ or *T*_2_ node-positive invasive breast cancer between 2008 and 2014 in the Netherlands. Data were retrieved from the population-based Netherlands Cancer Registry (NCR), which is a prospectively maintained database of all malignancies diagnosed in the Netherlands, based on notification by the Dutch nationwide pathology archive (PALGA) since 1989, containing information directly from patients’ medical records in all hospitals in the Netherlands. The use of these data was approved by the NCR Committee of Privacy.

Historically, all breast cancer patients underwent an ALND for axillary staging. The SLNB was officially incorporated in the Dutch guideline in 2004 [15]. Ever since the guidelines of 2008, it was recommended to perform sonographic evaluation of the axilla in all breast cancer patients with ultrasound-guided biopsies of the tumor and of suspicious axillary lymph nodes, irrespective of the palpability of the lymph node, prior to the SLNB [1, 16]. Histological biopsies of the breast tumor were performed using a 14-gauge or 18-gauge needle, whereas suspicious lymph nodes were biopsied for cytological analysis using a 21-gauge hollow needle. If pathological analysis showed that the ultrasound-guided biopsy was negative or inconclusive, patients underwent a SLNB. For the current analysis, only patients without clinically palpable lymphadenopathy (cN_0_) who had node-positive disease after an ALND were included. Patients with stage IV breast cancer, with clinical stage *T*_3_–*T*_4_ breast tumor according to the TNM classification [1], those receiving neoadjuvant systemic treatment, those with palpable axillary nodes (cN ≥ 1), and those who did not undergo an ALND were excluded.

### Data analyses

Clinical data included in the analysis were as follows: age, year of diagnosis, lateralization of the tumor, and type of surgery (mastectomy or breast conserving), and clinical TNM classification. Histopathological data collected on the tumor included tumor size according to the TNM classification [1], tumor morphology, tumor grade according to the Nottingham grading scale, and hormone and HER2 receptor status. Estrogen and progesterone receptor status were considered positive if 10% or more of the material contained the appropriate receptors. The presence of a multifocal tumor was also included in the analysis and was defined as tumors occurring in multiple sites in the breast. Information on whether or not patients had received an axillary ultrasound was not available in the NCR. Therefore, the group “UGLNB-positive patients” consisted of those patients who had a positive nodal status after an ALND, but did not undergo a previous SLNB. The total number of resected lymph nodes and the total number of positive lymph nodes included all nodes, either found after SLNB or after ALND. The total number of positive axillary lymph nodes was used to define the extent of nodal involvement, which was categorized as minimal nodal involvement (one or two positive nodes) or extensive nodal involvement (three or more positive lymph nodes), as proposed by the Z0011 trial.

In univariate analyses, the Chi-square test was used to compare differences in patient and tumor characteristics between patients who possibly had a positive UGLNB (UGLNB group) versus those with a positive SLNB (SLNB group). Survival analyses were conducted using the Kaplan–Meier method and a multivariate Cox regression analysis to calculate the Hazard Ratio (HR). A *p* value of ≤0.1 in univariate analysis was considered statistically significant and used as a threshold for inclusion of a covariate in the multivariate Cox regression analysis to establish whether the significant association between method of axillary staging and overall survival was influenced by these covariates. Overall survival was calculated from the date of diagnosis until death or last documentation. Follow-up was updated until December 2015. A *p* value of ≤0.05 in multivariate analysis was considered statistically significant.

## Results

**Fig. 1:**
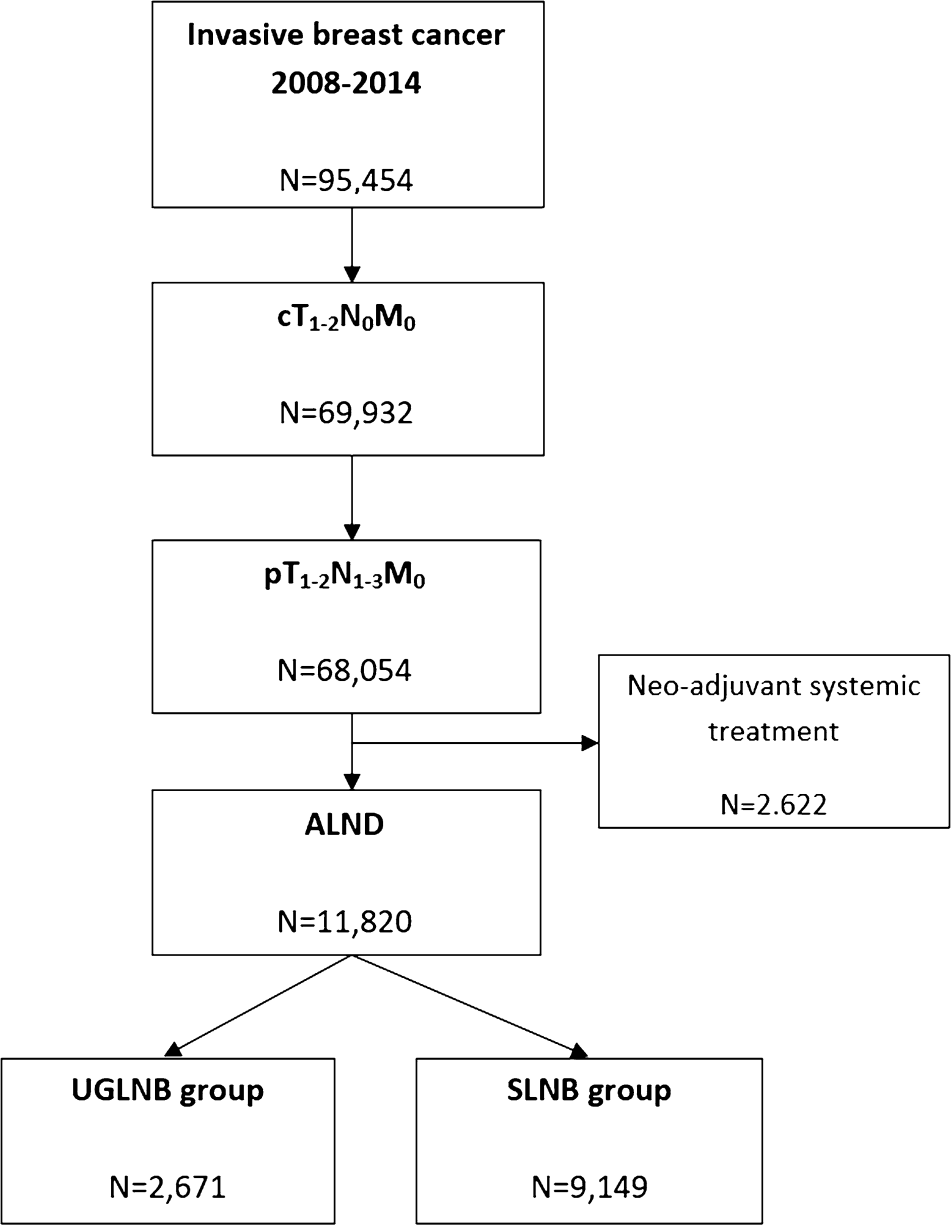
Flowchart of patient selection, using TNM classification [1], showing the inclusion of 2671 cases in the UGLNB group and 9149 cases in the SLNB group with positive lymph nodes after ALND. *ALND* axillary lymph node dissection, *SLNB group* patients with a positive sentinel lymph node biopsy, *UGLNB group* patients with a positive ultrasound-guided lymph node biopsy

From January 2008 until December 2014, a total of 95,454 patients were diagnosed with invasive breast cancer in the Netherlands. Their median age was 59 years, ranging from 21 to 97 years. After applying inclusion and exclusion criteria, as summarized in Fig. 1, a total of 11,820 patients had one or more positive lymph nodes and underwent an ALND. Of these patients, 9149 had a positive SLNB and the remaining 2671 were included in the UGLNB group.

### Differences in characteristics

Table 1 shows the distribution and the results of univariate analyses on patient and tumor characteristics between UGLNB-positive patients versus those with a positive SLNB. These results show a shift from node-positive diagnoses by ultrasound in the earlier years to a positive SLNB later on. Furthermore, analyses on patient and tumor characteristics showed that node-positive patients diagnosed by SLNB significantly differed from patients in the UGLNB group; the UGLNB group was older, was more often diagnosed in the period 2008–2011, had a larger tumor, had a poorly differentiated tumor, was more likely to have a negative hormone receptor status, had a multifocal tumor, had undergone a mastectomy, and was less likely to receive adjuvant radiation therapy or systemic therapy.

**Table 1:** Univariate analysis of patient and tumor characteristics in patients staged *pT*_1_–*T*_2_ with positive axillary lymph nodes identified by ultrasound versus sentinel node biopsy

Patient characteristics	UGLNB (*n* = 2671)	SLNB (*n* = 9149)	*p* value
Gender			0.741
Male	21 (0.8%)	78 (0.9%)	
Female	2650 (99.2%)	9071 (99.1%)	
Age			<0.001
Median [range]	63 [23–97]	58 [21–95]	
<50 years	590 (22.1%)	2305 (25.2%)	
50–69 years	1155 (43.2%)	5045 (55.1%)	
≥70 years	926 (34.7%)	1799 (19.7%)	
Year of diagnosis			<0.001
2008–2011	2246 (84.1%)	6788 (74.2%)	
2012–2014	425 (15.9%)	2361 (25.8%)	
Side of tumor			0.932
Right	1310 (49.0%)	4494 (49.1%)	
Left	1361 (51.1%)	4655 (50.9%)	
Type of surgery			<0.001
Breast conserving	649 (24.3%)	4464 (48.8%)	
Mastectomy	2022 (75.7%)	4685 (51.2%)	
Tumor stadium (TNM)			<0.001
pT1a	51 (1.9%)	88 (1.0%)	
pT1b	247 (9.2%)	801 (8.8%)	
pT1c	1082 (40.5%)	4222 (46.1%)	
pT2	1291 (48.3%)	4038 (44.1%)	
Morphology of tumor			<0.001
Ductal carcinoma	1970 (73.8%)	7116 (77.8%)	
Lobular carcinoma	351 (13.1%)	1097 (12.0%)	
Other types	350 (13.1%)	936 (10.2%)	
Tumor grade			<0.001
Grade 1	497 (18.6%)	1948 (21.3%)	
Grade 2	1235 (46.2%)	4414 (48.2%)	
Grade 3	838 (31.4%)	2518 (27.5%)	
Unknown	101	269	
ER status			<0.001
Negative	302 (11.3%)	579 (6.3%)	
Positive	2254 (84.4%)	8080 (88.3%)	
Unknown	115	490	
PR status			<0.004
Negative	578 (21.6%)	1327 (14.5%)	
Positive	1825 (68.3%)	6571 (71.8%)	
Unknown	787	1251	
HER2 status			0.474
Negative	2271 (85.0%)	7912 (86.5%)	
Positive	328 (12.3%)	1089 (11.9%)	
Unknown	72	148	
Multifocality			<0.001
No	1726 (64.6%)	7291 (79.7%)	
Yes	928 (34.7%)	1808 (19.8%)	
Unknown	17	50	
Total positive lymph nodes			<0.001
Median [range]	1 [1–29]	1 [1–34]	
1–2 nodes	2243 (84.0%)	7196 (78.6%)	
3 or more nodes	413 (15.4%)	1944 (21.2%)	
Unknown	15	36	
Radiotherapy			<0.001
No	1650 (61.8%)	3722 (40.7%)	
Yes	1021 (38.2%)	5427 (59.3%)	
Adjuvant systemic therapy			<0.001
No	647 (24.2%)	876 (9.6%)	
Yes	2024 (75.8%)	8273 (90.4%)	
Chemotherapy	284 (14.0%)	1109 (13.4%)	
Hormone therapy	833 (41.2%)	2468 (29.8%)	
Both	907 (44.8%)	4696 (56.8%)	

*UGLNB* ultrasound-guided lymph node biopsy, *SLNB* sentinel lymph node biopsy, *ER status* estrogen receptor status, *PR status* progesterone receptor status

### Overall survival

**Fig. 2:**
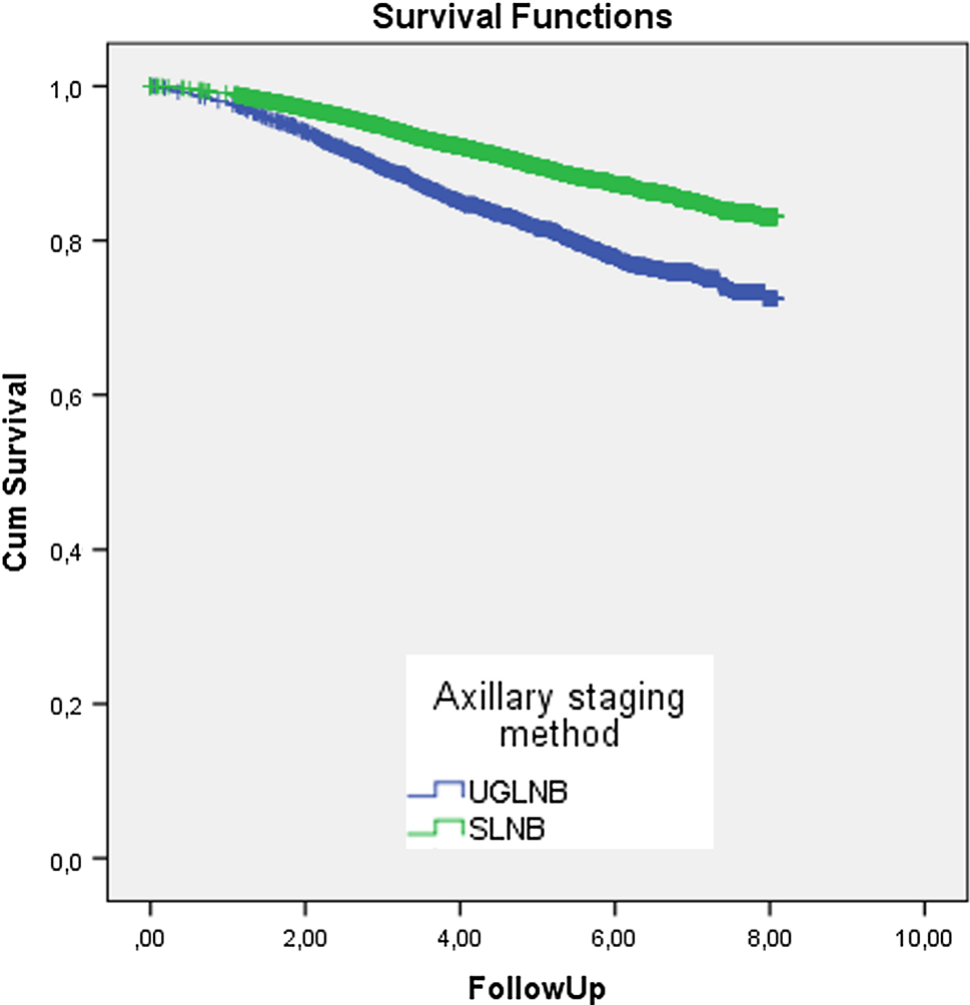
Kaplan–Meier curve of overall survival in years; SLNB group versus UGLNB group (*p* < 0.001). *SLNB group* patients with a positive sentinel lymph node biopsy, *UGLNB group* patients with a positive ultrasound-guided lymph node biopsy

The median follow-up time was 5 years, ranging up to 8 years. During follow-up, a total of 1542 patients (12.2%) died. The 5-year survival was 81.6% for the UGLNB group and 89.6% for the SLNB group (*p* < 0.001). Univariate Cox regression of the overall survival resulted in a hazard ratio (HR) of 1.82 (95% CI 1.64–2.02) for patients with a positive UGLNB compared to patients with a positive SLNB (Fig. 2).

Multivariate backward Cox regression analysis, adjusting for age at diagnosis, year of diagnosis, type of surgery, hormone receptor status, tumor morphology, tumor size, tumor grade, multifocality, number of positive lymph nodes, radiation therapy, and adjuvant systemic therapy, showed that a positive UGLNB remained significantly associated with a worse overall survival compared to a positive SLNB (HR = 1.38; 95% CI 1.23–1.56; *p* < 0.001) (Table 2).

**Table 2:** Significant results of multivariate backward Cox regression analyses on the association between method of axillary staging and overall survival

Characteristics	HR	95% CI	*p* value
Method of axillary staging			<0.001
SLNB	1		
UGLNB	1.38	1.23–1.56	
Age at diagnosis			<0.001
<50 years	1		
50–69 years	1.56	1.30–1.88	
≥70 years	4.39	4.07–5.88	
Tumor grade			<0.001
Grade 1	1		
Grade 2	1.21	1.03–1.43	
Grade 3	1.82	1.52–2.22	
ER status			0.001
Negative	1		
Positive	0.70	0.58–0.86	
PR status			0.001
Negative	1		
Positive	0.77	0.66–0.90	
Total positive lymph nodes			<0.001
1–2 nodes	1		
3 or more nodes	1.89	1.64–2.19	
Radiotherapy			0.004
No	1		
Yes	0.77	0.64–0.92	
Adjuvant systemic therapy			<0.001
No	1		
Yes	0.70	0.60–0.82	

*UGLNB* ultrasound-guided lymph node biopsy, *SLNB* Sentinel lymph node biopsy, *ER status* estrogen receptor status, *PR status* progesterone receptor status

In addition, we performed a sensitivity analysis excluding patients aged 70 years or older. These analyses showed that, during crude analyses, having a positive UGLNB was still significantly associated with a worse overall survival (HR = 1.21, 95% CI 1.03–1.43). However, multivariate cox regression analyses, adjusting for the aforementioned variables, show that method of axillary staging is no longer statistically significant (HR = 1.13, 95% CI 0.94–1.35).

## Discussion

The present nationwide, population-based study aimed to examine whether node-positive breast cancer patients found by UGLNB differ from those with a positive SLNB. It should be noticed that only patients without clinically palpable lymph nodes (cN_0_) and with small tumors (*pT*_1_–*T*_2_) were studied. It appears that pathologically node-positive patients selected by ultrasound had significantly worse disease characteristics and a worse overall survival compared to patients selected by SLNB, even after correction for possible confounders. However, this association was no longer existent when excluding patients aged 70 years or older. Thus, selection by axillary ultrasound appears to be an independent prognostic factor in patients younger than 70 years.

In a previous small single-center study, we examined this question in all breast cancer patients with and without clinically palpable lymphadenopathy. It was shown that patients with a positive UGLNB had worse disease characteristics and a higher axillary tumor burden [14]. This resulted in a worse disease-free (HR = 2.71; 95% CI 1.49–4.92) and overall survival (HR = 2.67; 95% CI 1.48–4.84) compared to patients with a positive SLNB. However, due to the small study population it was not possible to perform multivariate analyses to correct for confounding factors, as was done in the current study. The above-formulated question is of course most relevant for patients with a clinically negative axillary nodal status [17–22]. The current study has shown that having a positive UGLNB is associated with a worse survival in patients younger than 70 years, even after adjustment for known predictors of a worse prognosis, such as tumor size, tumor grade, hormone status, and number of positive lymph nodes [6, 11, 23–25]. Thus, being node positive after an UGLNB is a very important prognostic indicator in young breast cancer patients.

Furthermore, the present data demonstrate the ongoing shift in diagnostic work-up of the axillary lymph nodes over time. This has previously been described by Beek et al. [26] demonstrating the radical transformation in the work-up and management of the axilla in the southern Netherlands. Ever since 2004, Dutch guidelines recommend to perform a SLNB prior to an ALND for axillary staging. Although the axillary ultrasound was not yet a standard element of the diagnostic work-up, the guideline considered it to be useful for patient selection prior to the SLNB. In the guideline of 2008, the UGLNB was officially introduced as an additional diagnostic method preceding the SLNB [15, 16]. Therefore, since sonographic data were missing, patients who did not have a SLNB were included in the UGLNB group. The UGLNB group could therefore contain patients who underwent an immediate ALND, although this number should be limited.

Since the Z0011 trial has caused a paradigm shift in the management of invasive breast cancer, one might wonder whether the selection process of the axillary status may affect the applicability of its conclusions. The Z0011 demonstrated that in patients with *T*_1–2_ invasive breast cancer treated with breast-conserving therapy, including radiotherapy, and two or less positive sentinel nodes, the ALND could be omitted without affecting the prognosis [12]. However, some doubts were raised regarding the applicability of these Z0011 criteria in general practice, since this trial only included a selected group of node-positive patients detected by the SLNB [27, 28]. Multiple studies have shown a large variability in the proportion of patients who fulfill the inclusion criteria of the Z0011 trial, ranging from 9 to 75% [29–32]. This wide range is caused by differences in methods of inclusion and differences in the axillary work-up. Nevertheless, a number of health care centers have already implemented the Z0011 criteria into clinical practice, not only on patients who fit the Z0011 criteria, but also on other subgroups [26, 31, 33–37]. Unquestionably, application of the Z0011 criteria results in a substantial reduction of the number of unnecessary ALNDs within a specific group of node-positive patients [38, 39]. However, in patient categories which were not included in the Z0011 trial, such as patients who need a mastectomy or those with a positive UGLNB, ALND or axillary radiotherapy might still be necessary and recommended [27, 38]. These patients therefore remain at risk for the morbidity associated with these axillary treatment strategies.

Various studies have been performed on the clinical utility of UGLNB and on the applicability of the conclusions of the Z0011 trial on UGLNB-positive patients. Farrel et al. [39] examined the role of axillary ultrasound as a preoperative staging method. They performed subgroup analyses on patients who would be eligible for application of the Z0011 criteria if they were primarily staged by the SLNB. It was concluded that patients with a positive UGLNB had a larger axillary nodal burden and were therefore not suitable for application of the Z0011 criteria. On the other hand, patients with a negative UGLNB had minimal axillary nodal burden and were thus probably good candidates for treatment according to the conclusions of the Z0011 trial. In addition, Zgajnar et al. [40] compared node-positive patients who, prior to their SLNB, had either a negative physical examination or a negative UGLNB and concluded that patients with a negative ultrasound had a lower axillary tumor burden than patients who were classified as node negative after physical examination. Therefore, we advise to adhere to the use of UGLNB as a preoperative staging method, since the number of suspicious lymph nodes visualized during axillary ultrasound is predictive for the extent of nodal positivity which may still be relevant for determining the most optimal axillary treatment strategy [17, 18, 21, 26].

The results of the present study endorse that implementing the conclusions of the Z0011 trial should be done with caution, especially since international guidelines differ with regard to the axillary work-up. In the United States, an axillary ultrasound is only performed in patients with *clinically palpable lymph nodes*, whereas patients without palpable nodes will immediately undergo a SLNB [3]. On the contrary, in Europe basically *all breast cancer* patients receive an axillary ultrasound and will only undergo a SLNB in case of a negative or inconclusive UGLNB, irrespective of lymph node palpability [1, 2, 4]. Therefore, the patient cohorts underlying the studies, in which axillary nodal status plays a role, differ considerably between the US and Europe, due to the bias introduced by the differences with respect to axillary work-up.

The present retrospective study has some drawbacks. Specific data on the axillary sonographic evaluation were lacking, such as whether the UGLNB was performed at all and the sonographic results (like the number of visible nodes, number and results of the biopsy, etcetera). In order to distinguish patients with a positive UGLNB versus SLNB, we assumed that patients who had an ALND—but did not undergo a SLNB—had a positive UGLNB. As stated previously, the current UGLNB group may therefore also contain patients in whom an ALND was performed directly without prior axillary staging. However, this number should be limited, since both the UGLNB and the SLNB were a standard element of the axillary work-up, in the years included in this study. Furthermore, the follow-up of this study is limited. This results in missing data on recurrence rate and cause of mortality; therefore, the follow-up period should be extended and should focus on disease-free survival. It is also possible that not all confounders, such as comorbidities and body mass index, were identified due to the retrospective character of this study. Nevertheless, based on the high number of included patients in this study and the fact that these results confirm the findings of a previous study, to our opinion the conclusion of the present analysis should be considered valid and clinically relevant for the selection of node-positive breast cancer patients.

## Conclusion

This multicenter population-based study shows that young clinically node-negative breast cancer patients with pathological node positivity found by UGLNB have less favorable disease characteristics and a worse survival compared to patients selected to be node positive by a SLNB. This diagnostic selection process thus plays an important role when axillary treatment strategies are considered. Therefore, we conclude that the conclusions of the Z0011 trial cannot yet be applied to patients with a positive UGLNB.
